# The Role of the Cell Surface Heparan Sulfate Proteoglycan Syndecan-3 in Breast Cancer Pathophysiology

**DOI:** 10.3390/cells14201612

**Published:** 2025-10-17

**Authors:** Lena Habenicht, Nourhan Hassan, Nancy A. Espinoza-Sànchez, Jessica Oyie Sousa Onyeisi, Balázs Győrffy, Lars Hanker, Burkhard Greve, Martin Götte

**Affiliations:** 1 Department of Gynecology and Obstetrics, Münster University Hospital, Albert-Schweitzer Campus 1, 48149 Münster, Germany; nancyadriana.espinozasanchez@ukmuenster.de (N.A.E.-S.); jessicaoyie.Sousaonyeisi@ukmuenster.de (J.O.S.O.); lars.hanker@ukmuenster.de (L.H.); 2 Center for Molecular Medicine Cologne, University of Cologne, Robert-Koch-Straße 21, 50931 Cologne, Germany; nourhan.hassan@uk-koeln.de; 3 Biotechnology Department, Faculty of Science, Cairo University, Giza 12613, Egypt; 4 Department of Radiotherapy-Radiooncology, Münster University Hospital, Albert-Schweitzer Campus 1, 48149 Münster, Germany; greveb@uni-muenster.de; 5 Department of Bioinformatics, Semmelweis University, Tűzoltó utca 7-9, 1094 Budapest, Hungary; gyorffy.balazs@med.semmelweis-univ.hu

**Keywords:** syndecan-3, heparan sulfate, breast cancer, prognosis, proteoglycan, TFPI, MMP, cell proliferation, extracellular matrix

## Abstract

The heparan sulfate proteoglycan syndecan-3 (SDC3) is a critical regulator of cell–matrix interactions. While other syndecan family members contribute to the progression of multiple cancers, SDC3’s functional contributions to tumor biology remain largely unexplored. This study investigates the potential role of SDC3 in the pathogenesis of breast cancer. By conducting an in-silico analysis of publicly available datasets, including *TNM-plot*, *The Human Protein Atlas*, and *Kaplan–Meier Plotter*, we observed that SDC3 is upregulated in breast cancer tissue. Notably, high SDC3 expression correlates with improved relapse-free survival in breast cancer patients. In vitro experiments revealed that SDC3 depletion significantly impairs cell viability, cell-cycle progression, cell migration, and 3D-spheroid-formation in MDA-MB-231 and MCF-7 breast cancer cells. Furthermore, SDC3 depletion results in dysregulated gene expression of matrix metalloproteinases (*MMP1*, *MMP2*, *MMP9*) in MDA-MB-231 cells, and upregulation of E-cadherin (*CDH1*) and vascular endothelial growth factor A (*VEGFA*) in MCF-7 cells. Activation of proto-oncogene tyrosine-protein kinase Src was inhibited when SDC3 depletion was combined with tissue factor pathway inhibitor treatment. These findings demonstrate that breast cancer cell-derived SDC3 plays a pivotal role in tumor progression.

## 1. Introduction

Breast cancer is the second most common cancer and the fourth leading cause of cancer mortality worldwide. Among women, it is the most frequently diagnosed cancer and the leading cause of cancer-related deaths globally [1,2]. The disease encompasses a heterogeneous group of malignant tumors with diverse morphologies, biological behaviors, clinical presentations, and prognoses [3]. It is classified based on standardized pathomorphological criteria, including the histological type of tumor, histological degree of malignancy, stage according to the TNM classification, the expression of estrogen- and progesterone-hormone-receptors, the expression of human-epidermal-growth-factor-receptor (HER2), and the cellular proliferation index Ki-67 [4].

Recent research has highlighted the role of the extracellular matrix (ECM) in cancer progression [5]. The ECM is a complex network of macromolecules, that assembles into three-dimensional supramolecular structures with distinct biochemical and biomechanical properties [6,7]. ECM dynamics play key roles in physiological tissue development and homeostasis but may become dysregulated, once its composition is altered [6,7,8].

Proteoglycans are major structural components of the ECM and influence both cellular behavior and matrix properties through direct and indirect interactions with cytokines, growth factors, cell surface receptors, adhesion molecules, enzymes, and glycoproteins [9,10]. The most abundant subtype of proteoglycans are heparan sulfate proteoglycans (HSPGs), which are components of the cell surface and of the basement membrane that separates endothelial and epithelial cells from the underlying connective tissue [11]. Cell surface HSPGs serve as ECM receptors, and are classified into two major groups: syndecans, comprising four distinct members (SDC1 to SDC4), and glypicans, encompassing six members (GPC1 to GPC6) [12]. In healthy adult tissue, SDC1 is predominantly expressed in epithelia and plasma cells, syndecan-2 (SDC2) in endothelia and fibroblasts, SDC3 in neuronal and musculoskeletal tissue, while syndecan-4 (SDC4) is expressed heterogeneously [12,13]. Syndecans regulate multiple cellular signaling pathways due to their ability to act as coreceptors with signaling receptors, as well as endocytosis receptors [12]. In cancer, as well as in the cancer-surrounding stroma, syndecans can be abnormally expressed [14]. Syndecans play distinct roles in cancer pathogenesis depending on the cancer type and stage, either promoting or inhibiting cancer progression by influencing cell proliferation, migration, adhesion, invasion, and metastasis [14,15]. Several syndecan-targeting therapeutic approaches have been developed in recent years, including monoclonal antibodies, peptide inhibitors, biomolecule mimetics, and heparan sulfate mimetics or inhibitors that interfere with syndecan exosomal packaging [15].

SDC3 has previously been associated with the pathogenesis of ovarian cancer [16], pancreatic cancer [17], and renal cell carcinoma [18]. However, little is known about the potential role of SDC3 in breast cancer pathogenesis. To address our research question, we first performed an in-silico analysis utilizing large public datasets. Subsequently, we conducted in vitro experiments using the human breast cancer cell lines MDA-MB-231 and MCF-7, in which SDC3 downregulation was achieved by a single siRNA-sequence, and a pool of 30 sequences against SDC3.

## 2. Materials and Methods

### 2.1. The Human Protein Atlas, TNM Plot, and Kaplan–Meier Plot Analysis

The gene expression of SDC3 in healthy as well as pathological tissues was assessed using *The Human Protein Atlas* database (The Human Protein Atlas, https://www.proteinatlas.org, accessed 10 September 2024), which combines immunohistological tissue stainings, proteomics, and transcriptome analyses [19].

Additionally, the *TNMplot* database (TNMplot, https://tnmplot.com, accessed 10 September 2024) was utilized, which integrates gene array- and/or RNASeq-based public gene expression datasets from several sources (Gene Expression Omnibus of the National Center for Biotechnology Information (NCBI-GEO), The Cancer Genome Atlas (TCGA), Therapeutically Applicable Research to Generate Effective Treatments (TARGET), and The Genotype-Tissue Expression (GTEx)) [20]. To analyze SDC3 expression in breast cancer, the tools *Gene Expression Comparison, Gene Chip Data*, the gene symbol *SDC3,* and *breast tissue* were selected on the TNMplot website.

The open-access online tool *Kaplan–Meier Plotter* (*Kaplan–Meier Plotter*, www.kmplot.com, accessed 10 September 2024) [21] was used to conduct a survival analysis of SDC3 expression in breast cancer. *Kaplan–Meier Plotter* analysis was performed with *mRNA gene chip data.* The gene symbol *SDC3* (Affymetrix ID: 202898_at) and *relapse-free-survival* (RFS; *n* = 4929) was chosen on the website, the *median* was selected as the cut-off value, and *JetSet best probe* option was applied to ensure the selection of the optimal probe set [21].

### 2.2. Cell Culture

The human breast cancer cell lines MDA-MB-231, MCF-7, BT20, BT474, BT549, SK-BR3, HCC1806, MDA-MB-453, and MDA-MB-468 were obtained from ATCC/LGC Promochem (Teddington, Middlesex, UK). SUM-149 cells were purchased from BIOIVT (West Sussex, UK). MCF-7 cells were cultured in RPMI medium (PAN-Biotech GmbH, Aidenbach, Germany), with 10% fetal calf serum (FCS, PAN-Biotech GmbH, Aidenbach, Germany) and 1% penicillin/streptomycin (P/S) (Sigma-Aldrich, St. Louis, MO, USA), at 37 °C and 6.5% CO_2_. SUM-149 cells were cultured in Ham’s Nutrient Mixture F12 (Thermo Fisher Scientific, Waltham, MA, USA, cat. N66589) containing 5% FCS, 1% P/S, 5 µg of insulin (Sigma-Aldrich, St. Louis, MO, USA, cat. I9278), and 1 µg/mL hydrocortisone (Sigma-Aldrich, St. Louis, MO, USA, cat. H4001) in a Heracell 150i incubator (Thermo Fisher Scientific, Waltham, MA, USA Scientific) in a humidified atmosphere with 5% CO_2_ at 37 °C. All other cell lines were cultured in DMEM medium (Sigma-Aldrich, St. Louis, MO, USA), with 10% fetal calf serum (FCS) (PAN-Biotech GmbH, Aidenbach, Germany) and 1% P/S (Sigma-Aldrich, St. Louis, MO, USA), at 37 °C and 7.5% CO_2_.

### 2.3. siRNA Transfection

Prior to ntransfection, 3 × 10^5^ MDA-MB-231 or MCF-7 cells/well of a six-well plate cells were cultured in their respective routine culture media (see Section 2.2) for 24 h. Transfection with the Silencer Select Pre-designed siRNA (#s228527) (Ambion Life Technologies, Carlsbad, CA, USA), targeting exon 3 of human SDC3 mRNA (NM_014654.3), at 20 nM final concentration (referred to as ‘siRNA’), and the siPool (Lot. #SDC3-003) (siTools GmbH, Planegg-Martinsried, Germany), targeting 30 sequences of SDC3 (NCBI gene ID #9672), at 2.5 nM final concentration, to silence SDC3 (referred to as ‘siPool’), along with a Silencer Select Negative Control (ID#4390844) (Ambion Life Technologies, Carlsbad, CA, USA), and siPool Negative Control (Lot. #N000-c1-058) (siTools GmbH, Planegg-Martinsried, Germany) (referred to as ‘control’). The transfection mixture of 1 mL contained 0.84 mL Opti-MEM media (Cat. #31985070, Thermo Fisher Scientific, Waltham, MA, USA) and Dharmafect (Cat. #T-2001-01, Dharmacon, Lafayette, LA, USA), 0.08 mL SDC3 siRNA or SDC3 siPool or negative control siRNA or negative control siPool/Opti-MEM, and 0.08 mL 2.5% Dharmafect/Opti-MEM solution, according to the manufacturer’s instructions (Dharmacon). The knockdown efficiency was confirmed by quantitative real-time PCR (qRT-PCR).

### 2.4. Flow Cytometry

Forty-eight hours after transfection, cells were harvested with 2 mM EDTA/PBS, and 500,000 cells were used for antibody staining of SDC3 (Invitrogen, Karlsruhe, Germany). Recombinant Rabbit Monoclonal Antibody, 1:50, 24GB3890), with the secondary antibody (Invitrogen, Goat anti-Rabbit IgG, Alexa Fluor 488, 1:500, A11008), and corresponding isotype control (BD Pharmingen, San Diego, CA, USA, FITC Mouse IgG1, κ Isotype Control, 555909). All experiments were performed according to the manufacturer’s protocol. Measurement was performed on a flow cytometer (CyFlow space, Partec, Görlitz, Germany) and the data was visualized using FloMax software (Quantum Analysis, Münster, Germany).

### 2.5. Immunofluorescence

Cells (3 × 10^4^) were seeded on coverslips for 24 h, the cells were transfected, and 72 h after transfection, the cells were fixed with paraformaldehyde 4% for 10 min, and permeabilized with 0.2% Triton X-100 (Sigma-Aldrich, St. Louis, MO, USA) in PBS for 20 min. Cells were blocked for 1 h and then stained overnight at 4 °C with the primary antibody SDC3 (R&D Systems, Minneapolis, MN, USA; goat anti-Human Syndecan-3 Isoform 1 Antibody, 1:150, Cat. #: AF3539), and Vinculin (Sigma-Aldrich, Anti-Vinculin Antibody mouse monoclonal, 1:150, Cat. #: V9131). After that, cells were incubated for 30 min with the secondary antibodies: Donkey anti-Goat -IgG-Alexa fluor-488 (Invitrogen, 1:200, A-21427) and Rabbit anti-Mouse -IgG-Alexa fluor-555 (Invitrogen, 1:200, Cat. #: A-21427). Finally, nuclei were stained with DAPI (4′,6-diamidino-2-phenylindole dihydrochloride; Thermo Fisher Scientific, ref. D1306) for 25 min. Cells were observed and images were acquired using a confocal microscope (LSM 880 Carl Zeiss Microscopy GmbH, Jena, Germany) and analyzed using ZEN 2 software (Zeiss, Jena, Germany).

### 2.6. MTT Cell Viability Assay

The MTT assay for assessing cell viability was performed as previously described [16]. MDA-MB-231 and MCF-7 cells were cultivated on a 96-well-plate 48 h post-transfection. With a multichannel pipette, 100 μL of pre-warmed culture medium without phenol red (DMEM/RPMI with 10% FCS, 1% penicillin/streptomycin w/o phenol red; PAN-Biotech GmbH, Aidenbach, Germany) was first added to each well from row B to row H, except for row A. Next, 10,000 cells per well were added to the first two rows, A and B. A dilution series was created by suspending and transferring 100 μL of the cell suspension from row B to each of the lower rows, continuing until row G. The dilution series consisted of 10,000 cells/well in row A, 5000 cells/well in row B, 2500 cells/well in row C, 1250 cells/well in row D, 625 cells/well in row E, 312 cells/well in row F, and 156 cells/well in row G. The last row H contained a cell-free blank sample. Each sample was triplicated to generate mean values. The plate was incubated for 96 h at 37 °C. After incubation, the medium was removed and 20 μL of 3-(4,5-Dimethylthiazol-2-yl)-2,5-diphenyl-tetrazolium bromide (MTT) (Cat. # M2128-1G) at a concentration of 5 mg/mL was added. The cells were incubated for 4 h at 37 °C. Subsequently, 100 μL of MTT stop buffer containing 10% (*w*/*v*) sodium dodecyl sulfate (SDS) (Cat. # 3599286) and 50% (*v*/*v*) N,N-Dimethyl formamide), pH 4.7, was added per well to stop the reaction and dissolve the formazan crystals. The final incubation was performed for 24 h in the dark at room temperature. The photometric analysis was carried out at 570/650 nm with the ELISA-Reader VersaMax (Molecular Devices, San José, CA, USA).

### 2.7. Cell Cycle Analysis

Twenty-four h post-transfection, MDA-MB-231 and MCF-7 cells were serum-starved for 24 h. The cells were then harvested by Trypsin/EDTA (PAN-Biotech GmbH, Aidenbach, Germany) and subjected to the cell cycle analysis using DAPI (4′, 6′-diamidino-2-phenylindole) (Cat. #D9542, Sigma-Aldrich, St. Louis, MO, USA). The cell pellet was resuspended in 1 mL of DAPI solution (Cytecs, Münster, Germany). After 5 min of incubation at room temperature, the cells were analyzed using the CyFlow space flow cytometer (PARTEC, Münster, Germany), and the Guava easyCyte 3 HT reader (Merck Millipore; Billerica, MA, USA). Excitation of the cells was carried out with a 375 nm UV laser, and fluorescence emissions were measured at 455 nm in FL4.

### 2.8. Hanging Drop Assay for 3D Sphere Formation

The hanging drop method was utilized to generate three-dimensional spheroids [22]. MDA-MB-231 and MCF-7 cells were detached from 6-well plates using Trypsin/EDTA 48 h post-transfection. After washing and resuspending the cells in culture medium, a total of 12 drops, containing 20,000 cells per condition, were placed on the top lid of a petri dish. The bottom dish was filled with 10 mL PBS (Sigma-Aldrich, St. Louis, MO, USA), the petri dish was sealed, and spheroid formation was stimulated by gravity. The sealed petri dish was incubated at 37 °C. Images of the spheroids were documented on day 4 and day 7 of the experiments with an Axiovert (Zeiss, Jena, Germany) bright field light microscope (magnification 5×). Quantification of the core spheroid area was carried out with the program ImageJ (Rasband, W.S., ImageJ, U.S. National Institutes of Health, Bethesda, MD, USA, https://imagej.net/ij/, 1997–2018, accessed on 10 September 2024).

### 2.9. Cell Migration Assay

Twenty-four hours post-transfection, MDA-MB-231 and MCF-7 cells were harvested by Trypsin/EDTA, washed, resuspended in culture medium, and subjected to the cell migration assay. A total of 30,000 MDA-MB-231 cells and 75,000 MCF-7 cells were seeded per migration chamber (Cat. #353097, Corning Inc., Corning, NY, USA). The migration chambers were placed in the empty wells of a six-well plate and incubated for 24 h at 37 °C. Subsequently, the culture medium inside the chambers was removed, and 500 μL of culture medium without FCS was supplemented. Cell migration was induced by adding 750 μL of culture medium containing 5% FCS to each well of the 6-well plate. The plates were incubated for another 24 h at 37 °C. MDA-MB-231 cells were fixed and stained 24 h after the medium change, while the same procedures were performed on MCF-7 cells 72 h after the medium change. For staining, the chambers were removed from the six-well plate and placed in methanol (Merck KGaA, Darmstadt, Germany) for five minutes, then washed with PBS for one minute, and subsequently immersed in 1% toluidine blue staining solution (Cat. #89640, Sigma-Aldrich, St. Louis, MO, USA) for six minutes. The chambers were left to dry for 24 h, protected from light. Images were taken with the Axiovert (Zeiss, Jena, Germany) bright-field microscope (magnification 10×), and quantification of the experiments was performed with the program ImageJ (Rasband, W.S., ImageJ, U.S. National Institutes of Health, Bethesda, MD, USA, https://imagej.net/ij/, 1997–2018, accessed on 10 September 2024).

### 2.10. Wound-Healing Assay

Twenty-four hours post-transfection, MDA-MB-231 and MCF-7 cells and the respective controls were harvested and were seeded in a silicone culture-insert (ibidi culture-insert 2 well, ibidi GmbH, Martinsried, Germany) at a density of 2.5 × 10^5^ cells in 70 μL per well and incubated for 24 h. After that, cells were treated with 5 µg/mL of mitomycin C (Sigma-Aldrich, St. Louis, MO, US Cat. #M5353-0.2ML) for 2 h and were washed with PBS and the inserts were removed to achieve a reproducible cell-free gap. The cells were washed with 1× PBS before adding a standard medium. Pictures of the gaps were taken immediately after removing the inserts and 24 h afterwards. For each sample, three pictures at different gap locations were taken and analyzed with ImageJ Ver. 1.53. The wound area was calculated as follows: gap t(x h)/gap t(0 h) × 100%.

### 2.11. Total RNA Extraction and Quantitative Real-Time PCR

Total RNA extraction from SDC3-silenced MDA-MB-231 and MCF-7 cells was performed 72 h after transfection, using the InnuPREP RNA mini kit (Analytik Jena, Jena, Germany), according to the manufacturer’s protocol. Likewise, RNA was prepared from a panel of breast cancer cell lines under standard culture conditions to determine basal expression levels of SDC3. The High-Capacity cDNA Reverse Transcription Kit (Applied Biosystems, Foster City, CA, USA) was subsequently used for reverse transcription of mRNA (from 1 µg of total RNA) into cDNA. The SYBR Green qPCR Primer Assay (Biolegio, Nijmegen, The Netherlands) and Takyon ROX SYBR MasterMix blue dTTP (Eurogentec, Lüttich, Belgien) were used for quantitative real-time PCR (qRT-PCR) analysis of gene expression, using a 7300 real-time PCR detection system (Applied Biosystems, Foster City, CA, USA). Primer sequences are shown in Supplementary Table S1. Gene expression was documented using the 2^−∆∆Ct^ method after normalization to Actin gene expression as an internal control [23]. In our analysis, the relative gene expression was expressed as *fold change* and set in relation to the negative control.

### 2.12. Western Blotting

To corroborate the knockdown of SDC3, the MDA-MB-231 and MCF-7 cells were harvested 72 h post transfection. For TFPI treatment, after transfection, MDA-MB-231 and MCF-7 cells were serum-starved for 48 h and simultaneously treated with 50 ng/mL human recombinant TFPI (Lot: 6D30L4530, Sigma-Aldrich, St. Louis, MO, USA). Cell lysates were prepared using 200 µL/well of protein extraction buffer (Cell Signaling Technology, Danvers, MA, USA) 72 h post-transfection, and 48 h after TFPI treatment. Then, 15–30 μg of protein per group were separated by electrophoresis under reducing conditions and subsequently electrotransferred to a nitrocellulose membrane. The membrane was blocked with 5% skim milk in TBST buffer, pH 7.6. The primary antibodies (SDC3 R&D Systems, Human Syndecan-3 Isoform 1 Antibody, 1:150, Cat. #: AF3539; phospho-SRC, rabbit monoclonal, 1:1000, Cat. #2101, Cell Signaling Technology, Danvers, MA, USA; SRC, rabbit monoclonal, 1:1000, Cat. #2108, Cell Signaling Technology, Danvers, MA, USA; and GAPDH, mouse monoclonal, 1:5000, Santa Cruz Biotechnologies, Dallas, TX, USA) were diluted in 5% BSA in TBST buffer and incubated with the membranes overnight at 4 °C. The membranes were first probed with the phospho-SRC antibody and then reprobed with the additional antibodies after stripping of the membranes with 0.2 M glycine, pH 2.5. Following primary antibody incubation, the membranes were washed 3× with TBST. They were subsequently incubated with the secondary anti-goat, anti-rabbit or goat anti-mouse antibodies (1:5000, Merck KGaA, Darmstadt, Germany) in 5% skim milk for 1 h at room temperature. Signals were generated by a chemiluminescence ECL reaction with the SuperSignal West Pico PLUS Chemiluminescent Substrate (Thermo Fisher Scientific, Waltham, MA, USA) and documented with a Fusion SL Chemiluminescence Imaging System (Vilber Lourmat, Eberhardzell, Germany) device. Western blot images of three independent experiments were quantified by densitometric scanning using NIH Image J software release 1.54k (NIH, Bethesda, MD, USA), normalizing the signal of phospho-Src to total Src.

### 2.13. In-Silico Protein Interaction Network Analysis

STRING database version 12.0 (STRING, https://string-db.org, accessed 13 September 2024) was utilized to conduct an in-silico analysis of known and predicted protein–protein interaction networks related to SDC3. These interactions included both physical and functional interactions. Data from the STRING database was sourced from automated text mining of the scientific literature, computational interaction predictions from co-expression, conserved genomic contexts, as well as primary databases of interaction experiments [24]. To visualize the interactions of SDC3 with proteins of different signaling pathways, the protein name (*Sdc3*, *cell surface proteoglycan*) and organism (*homo sapiens*) were chosen on the STRING database website. The ten most significantly enriched GO terms (*p* < 0.05) were selected in the *biological processes*, *molecular function*, *cellular component*, and *KEGG pathways* categories. All the adjusted statistically significant values of the GO-terms were negative 10-base log-transformed.

### 2.14. Statistical Analysis

For the survival analysis, in the R statistical environment, we utilized the *Kaplan–Meier* Plotter database via the statistical package “survival”, to calculate *Kaplan–Meier* survival curves and the number-at-risk. Furthermore, the hazard ratio (and 95% confidence intervals) and log-rank P were calculated for each gene [25]. Furthermore, Microsoft Excel (Microsoft Corporation, Redmond, DC, USA) and GraphPad (GraphPad Software Inc., Boston, MA, USA) were used, to visualize the data. The unpaired two-tailed Student-*t*-test or unpaired *t*-test with Welch’s correction was utilized for two-way comparisons. The level of significance was set as *p* ≤ 0.05. Results with *p* ≤ 0.05 were considered statistically significant (*), while *p* ≤ 0.01 was described as very significant (**), and *p* ≤ 0.001 as highly significant (***). The graphs depict mean values, with error bars indicating the standard error of the mean (SEM). In each analysis, three independent biological experimental replicates were included (*n* = 3).

## 3. Results

### 3.1. SDC3 Is Overexpressed in Breast Cancer

**Figure 1:**
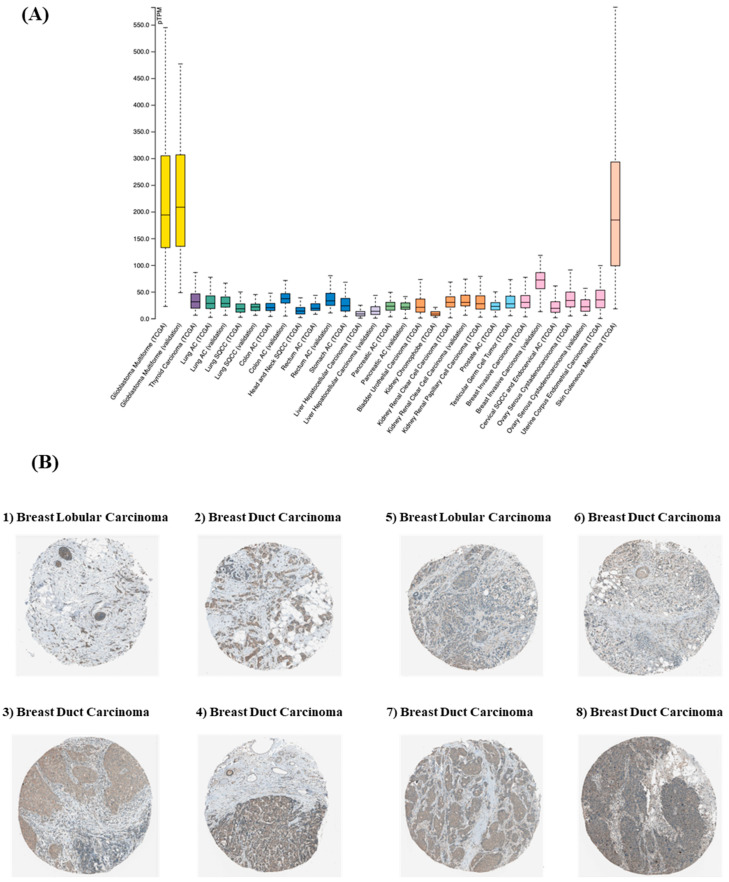
(**A**) Pan-cancer analysis of SDC3 expression across human malignancies. RNA-sequencing data from The Cancer Genome Atlas (TCGA) reveals median SDC3 expression levels across 31 cancer types, normalized as fragments per kilobase of exon per million mapped reads (FPKM). (**B**) SDC3 expression in breast cancer histological subtypes. Immunohistochemical staining of SDC3 in human breast tumor specimens presents as membranous and cytoplasmic staining that is primarily found in tumor cells, and less pronounced in the stroma. Scale bars are available at the original TCGA website.

As SDC3 has been implicated in the progression of ovarian [16], pancreatic [17], and renal cancers [18], we sought to determine whether the molecule contributes to the aggressive behavior of breast cancer cells, and if it could serve as a biomarker for the disease. The analysis of *The Human Protein Atlas* database (The Human Protein Atlas, https://www.proteinatlas.org, accessed 10 September 2024) revealed that from all gynecological tumors, SDC3 RNA expression was elevated in breast invasive carcinoma, as well as in other types of tumors, such as in glioblastoma and cutaneous melanoma (Figure 1A). *The Human Protein Atlas* furthermore included images of immunohistochemically stained breast cancer tissue samples, visualizing SDC3 protein expression. The staining was performed with the antibody #HPA017087 (Sigma-Aldrich, St. Louis, MO, USA), and reported as *low* or *medium*, based on conventional immunohistochemistry profiling of breast tissue, indicating that SDC3 is differentially expressed in breast cancer tissues (Figure 1B, Table 1). SDC3 expression was largely present within the tumor compared to the stroma, and showed a membranous and cytoplasmic staining pattern.

**Figure 2:**
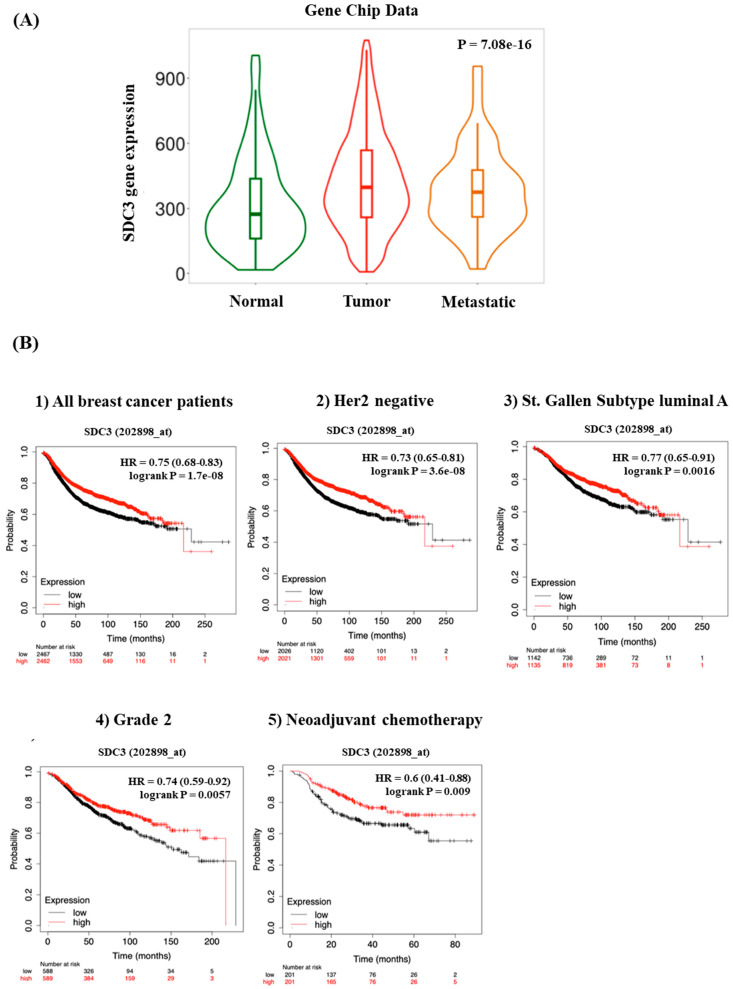
SDC3 is highly expressed in tumors and its expression correlates with better survival. (**A**) SDC3 expression varies across breast cancer progression stages. Gene chip analysis reveals significantly increased SDC3 mRNA levels in primary tumors and metastatic lesions, compared to normal breast tissue. (**B**) SDC3 expression is associated with the relapse-free survival (*RFS*) of breast cancer patients. *Kaplan–Meier* relapse-free survival curves are plotted based on the following: all breast cancer patients (*n* = 4929), HER2 negative status (*n* = 4047), St. Gallen luminal A status (*n* = 2277), Grade 2 (*n* = 1177), and neoadjuvant chemotherapy (*n* = 402). Log-rank *p* values and hazard ratios (HRs; 95% confidence interval in parentheses) are shown.

The open-access tool TNM-plot (TNMplot, https://tnmplot.com, accessed 10 September 2024) was used, to compare SDC3 gene expression in normal breast tissue, breast cancer tissue, and metastatic tissue (Figure 2A). TNM-plot analysis of gene chip data of normal tissue (*n* = 242), breast cancer tissue (*n* = 7569), and metastatic tissue (*n* = 82) demonstrated that SDC3 RNA was significantly upregulated in breast cancer tissue (Dunn-test *p* = 5.45 × 10^−17^) compared to normal tissue. However, SDC3 RNA expression was not significantly altered in metastatic tissue (Dunn-test *p* = 1.28 × 10^−1^) in relation to normal tissue.

### 3.2. SDC3 Expression Affects the Prognosis and Survival of Breast Cancer Patients

To investigate, whether SDC3 expression influences the relapse-free survival (*RFS*) of breast cancer patients, a *Kaplan–Meier Plotter* analysis was conducted, using the *KMplot* website (KMplot, https://kmplot.com/analysis/, accessed 10 September 2024). All breast cancer samples included in the analysis were stratified by receptor status, molecular subtypes, lymph node status, grade, and treatment (Figure 2B, Table 2). Our *Kaplan–Meier Plotter* analysis revealed that high SDC3 RNA expression levels served as a protective factor for the relapse-free survival of breast cancer patients overall. This correlation applied to Her2-negative-, St. Gallen subtype luminal A-, St. Gallen subtype luminal B-, St. Gallen subtype basal-, PAM50 subtype luminal B-, PAM50 subtype basal-, lymph node-negative, and Grade 2-tumors. High SDC3 expression was, furthermore, associated with better relapse-free survival in breast cancer patients following neoadjuvant chemotherapy.

Table 2 shows the HR (Hazard ratio) and *p* values of SDC3 for the RFS from the KM-Plot analysis. The analysis was performed based on general analysis and the stratification by receptor status, molecular subtypes, lymph node status, grade, and treatment. Bold typing of *p*-values indicates a significant association (*p* < 0.05).

### 3.3. SDC3 RNA Expression Varies in Breast Cancer Cell Lines of Different Classification

**Figure 3:**
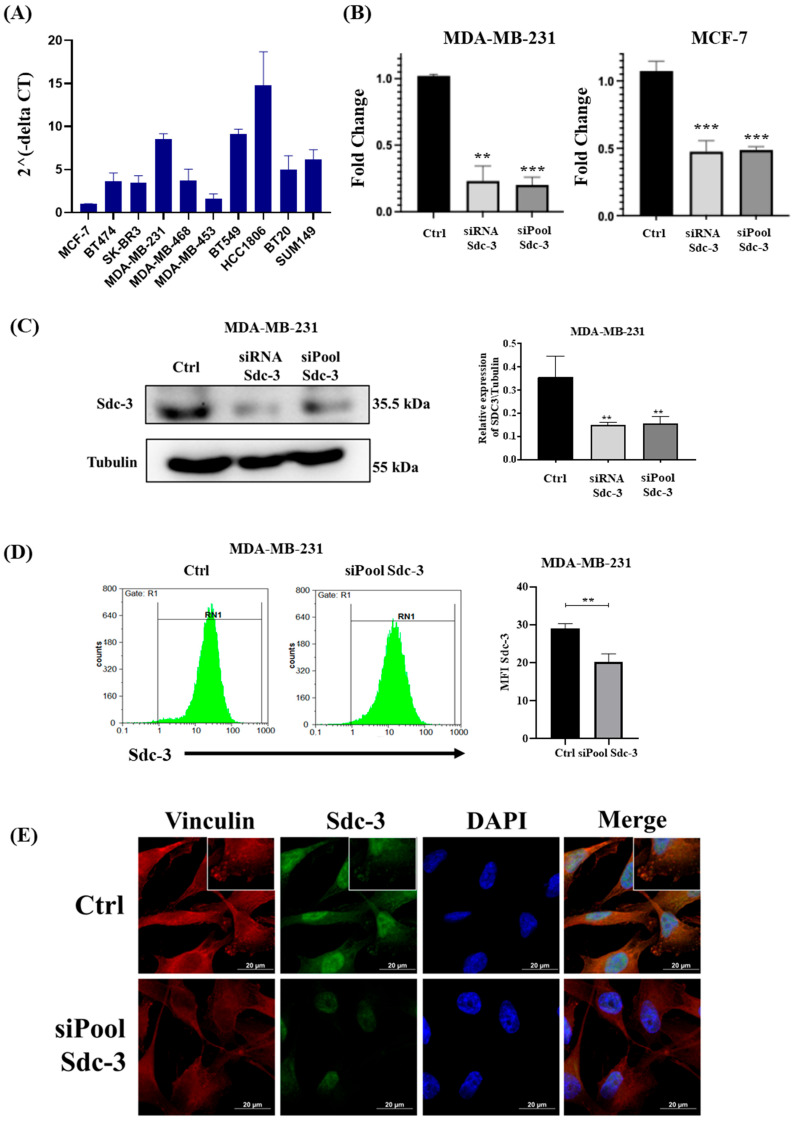
SDC3 is expressed differentially in several breast cancer cell lines and alters their metabolic activity and cell cycle. (**A**) Relative gene expression of SDC3 was quantified by qRT-PCR in 10 different breast cancer cell lines, representative of the luminal A (MCF-7), luminal B (BT474), HER2-positive (SKBR3, MDA-MB 453), triple-negative A (MDA-MB 468, HCC1806, BT20), and triple-negative B (MDA-MB-231, BT549, SUM149) subtype. Individual experiments were normalized against β-Actin and the relative expression was represented by 2-ΔCt. (**B**) SDC3 is downregulated in MDA-MB-231, and MCF-7 cells following SDC3 siRNA- and siPool-transfection. SDC3 knockdown was confirmed by qRT-PCR. (**C**–**E**) The downregulation of SDC3 was further assessed by Western blot (**C**), flow cytometry (**D**), and immunostaining for SDC3 (green fluorescence), vinculin (red), and nuclear staining with DAPI (blue) (**E**). The analysis revealed a reduced expression of SDC3 in KD cells (**D**), as well as its constitutive cellular localization. In control cells, SDC3 was predominantly localized in the cytoplasm, nucleus, and in focal adhesion-like structures. In contrast, KD cells exhibited a loss of focal adhesion-like and cytoplasmic staining of SDC3 (**E**). Inserts highlight magnified regions of individual cells, indicating SDC3 expression and vinculin in structures resembling focal adhesions of control cells and the absence of these structures in KD cells. *p* ≤ 0.01 was described as very significant (**), and *p* ≤ 0.001 as highly significant (***). The graphs depict mean values, with error bars indicating the standard error of the mean (SEM). Three independent experimental replicates were included (*n* = 3); original magnification 400×.

qRT-PCR analysis was performed, to measure the gene expression levels of SDC3 in seven different human breast cancer cell lines. The cell lines represent different molecular breast cancer subtypes, including the luminal A (MCF-7), luminal B (BT474), HER2-positive (SKBR3, MDA-MB 453), triple-negative A (basal-like) (MDA-MB 468, HCC1806, BT20), and triple-negative B (normal-like/claudin-low) (MDA-MB-231, BT549, SUM149 subtype [26]. Our qRT-PCR analysis demonstrated that SDC3 gene expression levels varied substantially across the breast cancer cell lines investigated (Figure 3A). For our additional functional studies, we selected the widely used MDA-MB-231 as one of the highest, and MCF-7 as one of the lowest SDC3-expressing cell lines [27]. MDA-MB-231 cells model the *triple-negative* breast cancer subtype, which is characterized by the absence of estrogen- (ER-), progesterone- (PR-), and Her2- (Her2-) receptors. MCF-7 cells are classified as the *luminal A* breast cancer subtype, as they express estrogen (ER+), and progesterone receptors (PR+), but not Her2-receptors (Her2-) [26].

### 3.4. SDC3 Depletion Affects the Metabolic Activity and Cell Cycle of Human MDA-MB-231 and MCF-7 Breast Cancer Cells

Building on our in-silico analysis, which suggested a potential role of SDC3 in breast cancer pathogenesis, we conducted in vitro experiments with SDC3-depleted human MDA-MB-231 and MCF-7 breast cancer cells. We studied the impact of SDC3 downregulation in vitro using the siRNA and siPool knockdown approach, which were quantified by qRT-PCR and analyzed in relation to the cells transfected with the negative control (Figure 3B). In MDA-MB-231 cells, we observed a significant downregulation of approx. 80% of SDC3 expression, with both SDC3 siRNA (*p* ≤ 0.01) and SDC3 siPool (*p* ≤ 0.001) knockdown approaches. In MCF-7 cells, we detected a significant downregulation of approx. 50% of SDC3 expression, in both SDC3 siRNA (*p* ≤ 0.001) and SDC3 siPool (*p* ≤ 0.001) transfected cells. By Western blot, flow cytometry, and immunofluorescence (IF) analysis, we corroborated the downregulation of SDC3 at the protein level (Figure 3C–E). Interestingly, the analysis showed that in control cells, SDC3 was present in focal adhesion-like structures, the cytoplasm, and the nucleus (Figure 3E). Focal adhesion staining of SDC3 disappeared (Figure 3E, see magnified images), and cytoplasmic and nuclear staining was reduced in SDC3-KD cells, under our experimental conditions. We also stained the cells with the focal adhesion constituent Vinculin, which is a major regulator of cell adhesion, and we observed co-expression of vinculin with SDC3, corroborating that SDC3 is also expressed in focal adhesion-like structures (Figure 3E, see dot like staining in membrane protrusions in magnified images). For the first time, we were able to demonstrate that SDC3 is localized in focal adhesion-like structures in breast cancer cells.

**Figure 4:**
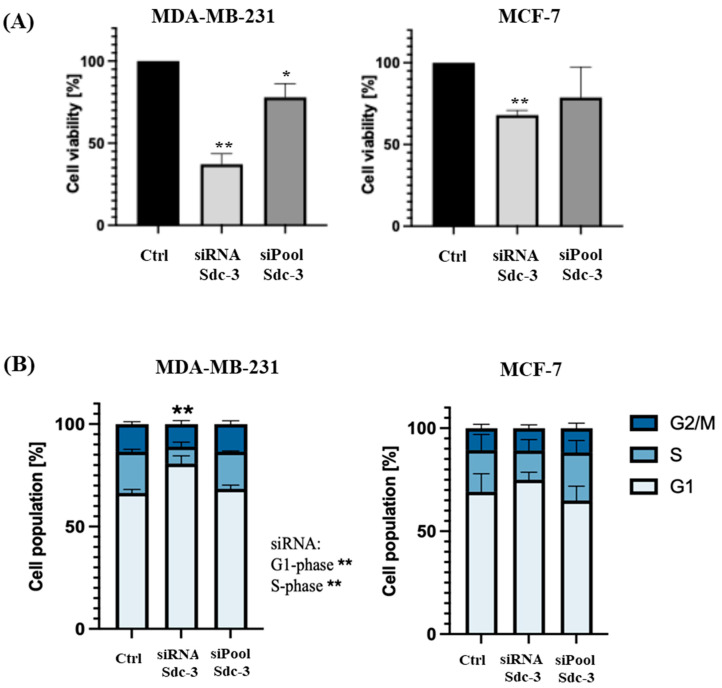
SDC3 alters the metabolic activity and cell cycle of breast cancer cells. (**A**) SDC3 depletion affects the metabolic activity of MDA-MB-231 and MCF7 breast cancer cells. Breast cancer cells were subjected to the metabolic MTT assay following transfections with the siRNA and siPool. (**B**) SDC3-knockdown promotes cell cycle progression in MDA-MB-231 cells. Cell cycle phase composition was measured employing DAPI staining and flow cytometry after SDC3 depletion. *p* ≤ 0.05 was considered statistically significant (*), while *p* ≤ 0.01 was described as very significant (**). The graphs depict mean values, with error bars indicating the standard error of the mean (SEM). Three independent experimental replicates were included (*n* = 3).

We then analyzed the effect of SDC3 depletion on cell viability, utilizing the metabolic MTT assay (Figure 4A). The analysis revealed that the metabolic activity of MDA-MB-231 cells following siRNA-mediated SDC3 knockdown was significantly decreased by approx. 63% (*p* ≤ 0.01), while it was reduced by approx. 22% (*p* ≤ 0.05) following SDC3 knockdown with the siPool. A similar effect was observed in MCF-7 cells, where the metabolic activity was significantly decreased by approx. 32% (*p* ≤ 0.01) upon siRNA-mediated SDC3 knockdown. A non-significant reduction in metabolic activity was observed following SDC3 depletion with the siPool.

To investigate the potential role of SDC3 on the cell cycle, a flow cytometry analysis with transfected MDA-MB-231 and MCF-7 cells was performed (Figure 4B). Significant changes in the G1- and S-phase of the cell cycle were observed in MDA-MB-231 cells transfected with the SDC3 siRNA, as significantly more cells accumulated in the G1-phase (80.7%; *p* ≤ 0.01), while significantly fewer cells were detected in the S-phase (8.2%; *p* ≤ 0.01), compared to the negative control (G1-phase: 66.4%; S-phase: 20.0%). The G2/M-phase was not altered in MDA-MB-231 cells following transfection with the SDC3 siRNA. By contrast, MDA-MB-231 cells transfected with the siPool did not exhibit alterations of the cell cycle, and the proportions of cells detected in the G1-, S-, and G2/M phases were similar to those of the negative control. The cell cycle of MCF-7 cells transfected with the SDC3 siRNA and siPool was not significantly altered in our analysis.

### 3.5. SDC3 Depletion Affects Three-Dimensional Spheroid Growth of Human MDA-MB-231 and MCF-7 Breast Cancer Cells

**Figure 5:**
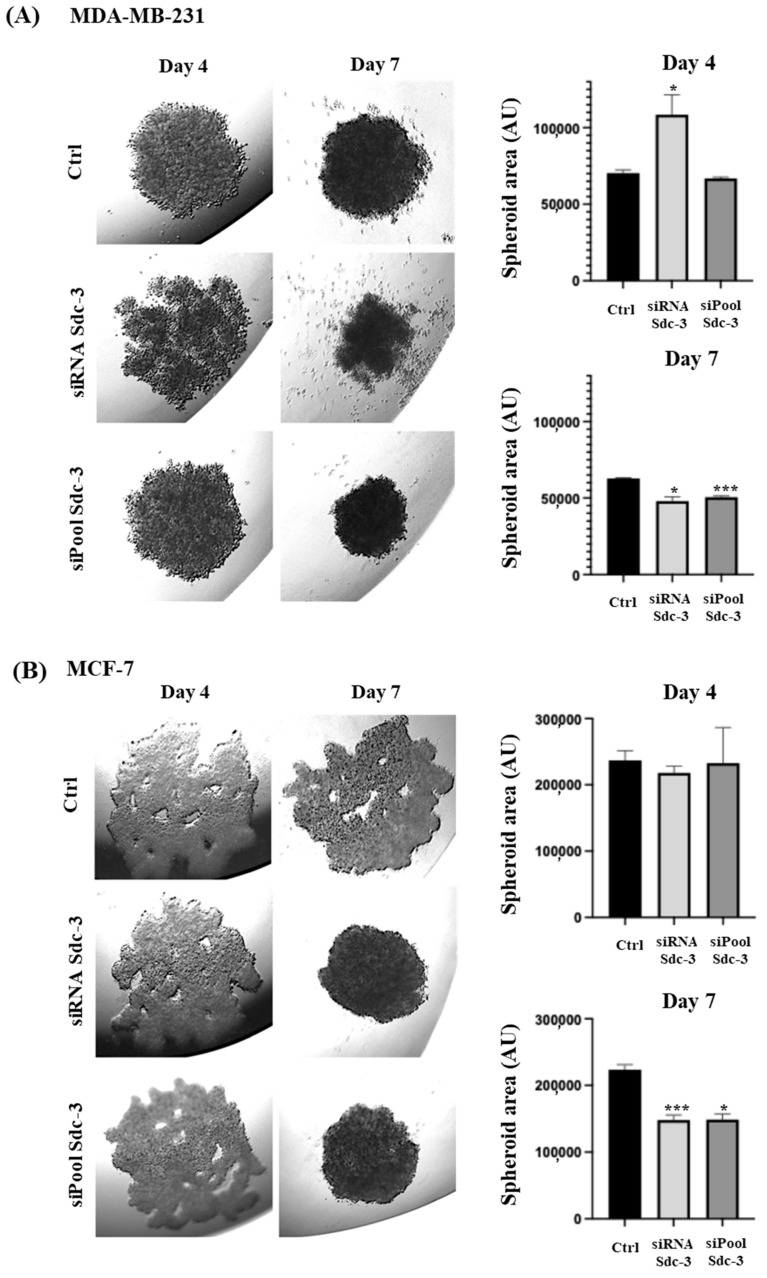
SDC3 depletion affects the growth of 3D spheroids in MDA-MB 231 and MCF-7 cells. Representative pictures of the hanging drop cultures are presented in the left panels and quantitative analysis in the right panels. Spheroid area was quantified using NIH Image J software and expressed as arbitrary units (AU). (**A**) MDA-MB-231 cells were transfected with the negative control, SDC3 siRNA, and SDC3 siPool, and subjected to the hanging drop assay. (**B**) MCF7 cells were transfected with the negative control, SDC3 siRNA, and SDC3 siPool, and subjected to the hanging drop assay. *p* ≤ 0.05 was considered statistically significant (*), and *p* ≤ 0.001 as highly significant (***). The graphs depict mean values, with error bars indicating the standard error of the mean (SEM). Three independent experimental replicates were included (*n* = 3).

We analyzed the effect of SDC3 downregulation on the growth of three-dimensional (3D) spheroids in a hanging drop assay, as a readout of stem cell activity [22,28,29]. MDA-MB-231 cells transfected with the negative control formed compact, round spheroids from day 4 until day 7. In comparison, SDC3 siRNA-transfected MDA-MB-231 cells formed less cohesive spheroids that exhibited loose, irregular shapes on day 4. By day 7, the spheroids started to dissolve, single cells drifted away from the edges, and spread throughout the culture medium. Quantification of the core spheroid area showed that siRNA-mediated SDC3 depletion resulted in a significant increase (*p* ≤ 0.05) in sphere size on day 4, while a significant decrease (*p* ≤ 0.05) was visible on day 7. Interestingly, MDA-MB-231 cells transfected with the siPool formed compact, round spheroids from day 4 until day 7 and did not disintegrate in a similar manner as MDA-MB-231 cells transfected with the SDC3 siRNA. On day 4, the sphere size of the SDC3 siPool-transfected cells was non-significantly altered; however, a significant decrease (*p* ≤ 0.001) was detected by day 7 (Figure 5A).

MCF-7 cells transfected with the negative control formed spheroids of irregular shapes with several side extensions and holes from day 4 until day 7. This morphology was also observed in MCF-7 cells transfected with the SDC3 siRNA on day 4. However, by day 7, SDC3 siRNA-transfected MCF-7 cells formed spheroids of round, compact shapes. The sphere size of MCF-7 cells transfected with the SDC3 siRNA was non-significantly altered on day 4 and significantly decreased (*p* ≤ 0.001) on day 7, in comparison to the negative control. A similar trend was observed for MCF-7 cells transfected with the SDC3 siPool. On day 4, the spheroids of siPool-transfected MCF-7 cells exhibited irregular shapes with several side extensions and holes, and by day 7, the spheroids were round and compact. The sphere size of MCF-7 cells transfected with the SDC3 siPool was non-significantly altered on day 4, by day 7, it was significantly decreased (*p* ≤ 0.05) compared to the negative control (Figure 5B).

### 3.6. SDC3 Depletion Affects Cell Migration of Human MDA-MB-231 and MCF-7 Breast Cancer Cells

**Figure 6:**
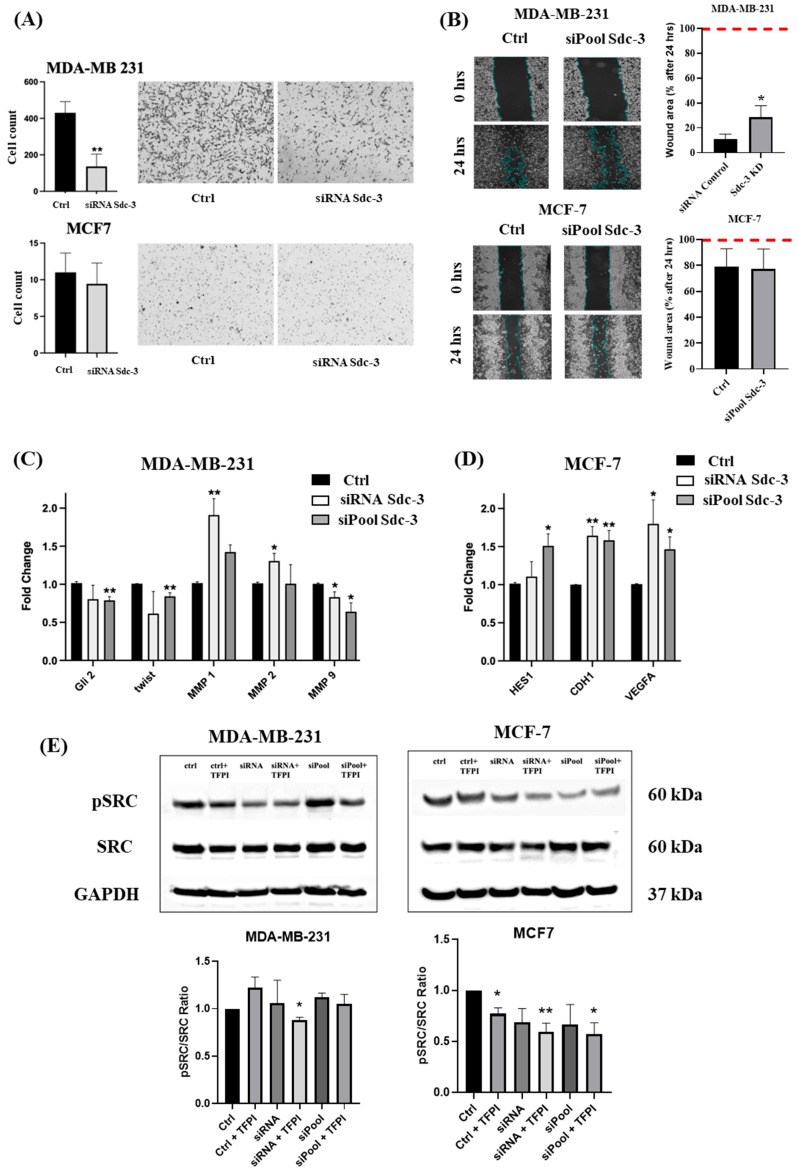
(**A**) The migration of MDA-MB-231 cells was significantly decreased in transwell migration assays (10× magnification) and the (**B**) wound area was significantly increased in ibidi chamberwound closure assays following SDC3 knockdown, in comparison to the negative control. MCF-7 cells displayed little changes in migratory behavior or in wound area upon SDC3 depletion. Red line indicates maximum wound closure. 10× magnification.(**C**,**D**) SDC3 regulates the expression of different potential effector genes. SDC3 depletion was achieved by siRNA- and siPool-mediated knockdown of SDC3 in MDA-MB-231 (**C**), and MCF-7 (**D**) cells. The expression of target genes associated with notch-signaling (*HES1*), hedgehog-signaling (*Gli2*), epithelial-to-mesenchymal-transition signaling (*Twist*, *CDH1*), matrix-metalloproteinase signaling (*MMP1*, *MMP2*, *MMP9*), and vascular endothelial growth factor signaling (*VEGF-A*) was visibly altered (**E**) The effect of SDC3 depletion on the potential activation of Src proto-oncogene tyrosine-protein kinase (pSRC), as well as expression of its total form (*SRC*) was examined using Western blot analysis. Upper panels = representative Western blots. Lower panels = densitometric quantification of three independent experiments. *p* ≤ 0.05 was considered statistically significant (*), while *p* ≤ 0.01 was described as very significant (**). The graphs depict mean values, with error bars indicating the standard error of the mean (SEM). Three independent experimental replicates were included (*n* = 3).

The cell migration and wound healing assay was conducted with siRNA-transfected MDA-MB-231 and MCF-7 cells. The migration of MDA-MB-231 cells was significantly decreased by 68% (*p* ≤ 0.01) following SDC3 knockdown, in comparison to the negative control. MCF-7 cells displayed little migratory behavior in general, and cell migration was not significantly altered upon SDC3 knockdown compared to the negative control (Figure 6A). When we performed a wound closure assay in the presence of mitomycin C (eliminating a possible confounding influence of cell proliferation), the wound healing area of SDC3 knockdown MDA-MB-231 cells was significantly increased (*p* ≤ 0.05) in comparison to the negative control, which indicates less migration capacity (Figure 6B). On the contrary, no changes between control and the MCF-7 SDC3 knockdown cells were observed (Figure 6B).

### 3.7. SDC3 Depletion Affects the RNA Expression of Target Genes Associated with Relevant Signaling Pathways in Breast Cancer

qRT-PCR was performed with transfected MDA-MB-231 and MCF-7 cells to explore the effects of SDC3 depletion on the expression of several target genes associated with relevant signaling pathways in breast cancer. The gene expression of SDC1 (*Sdc1*) and SDC4 (*Sdc4*) was evaluated in SDC3-depleted cells to determine potential compensatory expression changes. Moreover, the expression of target genes associated with notch-signaling (*Notch1*, *HES1*), wnt-signaling (*WNT5A*, *TCF7L1*), hedgehog-signaling (*Gli1*, *Gli2*, *Gli3*), epithelial-to-mesenchymal-transition (*Twist*, *Snail1*, *Snail2*, *CDH1*, *Vimentin*), matrix-metalloproteinases (*MMP1*, *MMP2*, *MMP9*), angiogenesis (*VEGF-A*), inflammation (*IL-8*), basement membranes (*COL4A2*), and CD44-signaling (*CD44*) was investigated. These target genes were chosen for our analysis, as previous studies have shown the involvement of other syndecan family members in these pathways [13,15,16,30,31,32,33].

In MDA-MB-231 cells, the most consistent changes in gene expression were observed for *Gli2*, *Twist*, *MMP1*, *MMP2*, and *MMP9* following SDC3 knockdown with the siRNA and siPool. While *Gli2*, *twist*, and *MMP9* were downregulated upon SDC3 knockdown, an upregulation was observed for *MMP1* and *MMP2* (Figure 6C, Supplementary Figure S1A). In MCF-7 cells, siRNA- and siPool-mediated SDC3 knockdown resulted in consistent upregulation of *HES1*, *CDH1*, and *VEGF-A* (Figure 6D, Supplementary Figure S1B). Notably, SDC3 knockdown with the siPool led to an upregulation of SDC4 in MCF7 cells (Supplementary Figure S1B).

MDA-MB-231 cells exhibit mesenchymal traits and high aggressiveness, whereas MCF-7 cells display an epithelial phenotype with lower aggressiveness [34]. Following the downregulation of SDC3, a reduction in the expression of EMT-associated genes was observed in MDA-MB-231 cells, indicating a potential phenotypic shift toward a less mesenchymal and less aggressive state (Figure 6A,B). In MCF-7 cells, an upregulation of *CDH1* expression was detected, which aligns with the maintenance of an epithelial phenotype and the absence of significant changes in invasiveness (Figure 6A,B). These results suggest that SDC3 may influence the aggressiveness of breast cancer cells by modulating the expression of EMT-markers.

### 3.8. SDC3 Depletion and TFPI Treatment Synergistically Affect the Activation of SRC of Human MDA-MB-231 and MCF-7 Breast Cancer Cells

To further evaluate potential mechanisms by which SDC3 influences cell viability, cell-cycle phases, stemness-associated factors, and cell migration mainly in MDA-MB-231 and in MCF-7 breast cancer cells, we examined the effect of SDC3 downregulation on the potential activation of Src proto-oncogene tyrosine-protein kinase (pSRC), as well as on the expression of its total form (*SRC*). Src has previously been associated with SDC3 function in a neurobiological context [35]. Moreover, Src is a key regulator of several oncogenic signaling pathways and has been implicated in promoting tumor angiogenesis, metabolic reprogramming, and metastasis in various cancers [36,37,38,39]. Since previous studies have demonstrated that another member of the syndecan family, SDC1 modulates the TF pathway in breast cancer [40], and since SDC3 is required for localization of issue-factor-pathway-inhibitor (TFPI) to endothelial-, smooth muscle-, and breast cancer cells [41], we investigated the effect of SDC3 depletion and TFPI treatment on Src activation.

Western blot analysis was performed with MDA-MB-231 and MCF-7 cells, following transfection with the negative control, SDC3 siRNA, and SDC3 siPool, and additional incubation with TFPI (Figure 6E).

Densitometric analysis of three independent experiments revealed that phosphorylation of Src has significantly inhibited both in MCF-7 and MDA-MB-231 cells when SDC3 siRNA knockdown was combined with TFPI treatment. In MCF-7-cells, TFPI treatment alone was sufficient to inhibit Src phosphorylation. Neither SDC3 depletion nor TFPI treatment reduced the expression of total Src. (Figure 6E).

### 3.9. STRING Functional Enrichment Analysis

**Figure 7:**
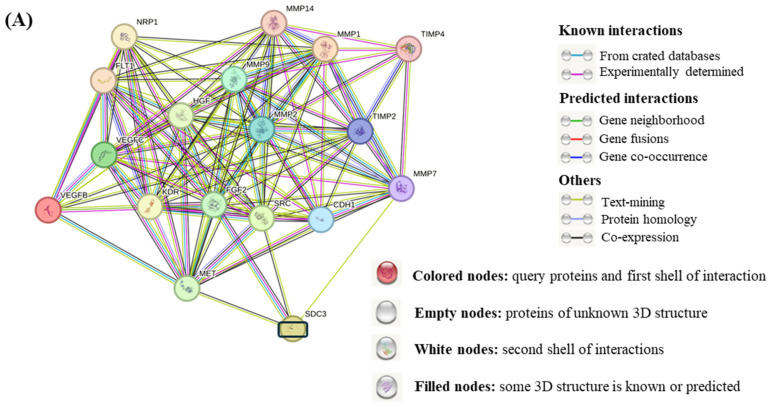

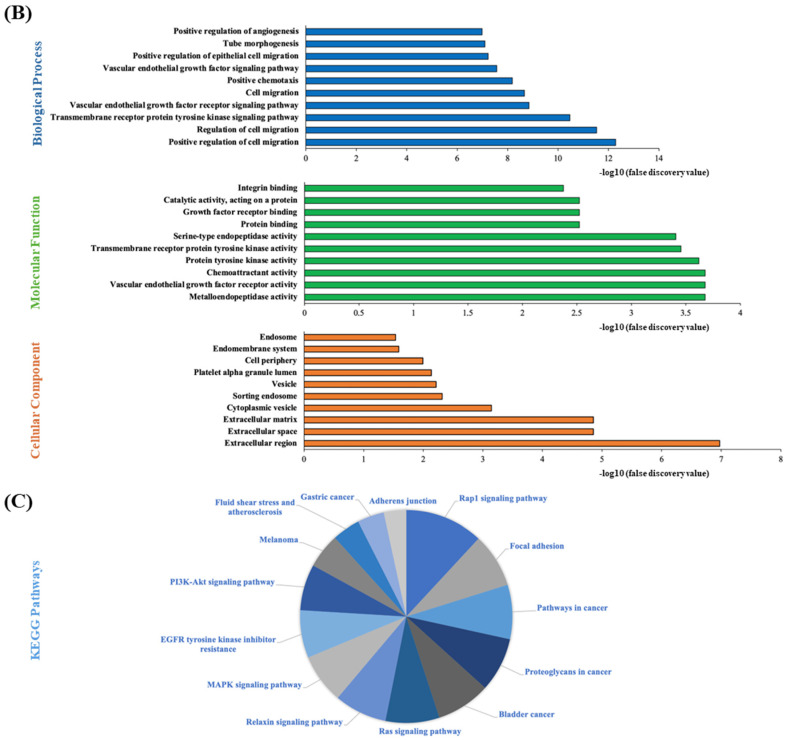
Functional analysis of SDC3 using STRING database. (**A**) STRING database output revealed direct connections between SDC3, *SRC*, *FGF2*, and *MMP7.* Further indirect associations were established between SDC3 and *FLT1*, *KDR*, *VEGFB*, *VEGFC*, *CDH1*, *MMP1*, *MMP2*, *MMP9*, and *MMP14*, as well *TIMP2* and *TIMP4*. (**B**) In the gene ontology (GO) analysis, the 10 most significantly (*p* < 0.05) enriched GO terms were included in the biological process (blue), molecular function (green), and cellular component (orange) categories. X-axis: −log10 (false discovery value) (**C**) KEGG-pathway analysis depicts several interconnections between SDC3 and relevant signaling pathways in cancer.

To investigate the molecular interactions between SDC3 and key proteins involved in signaling pathways in cancer, we performed an in silico functional interaction analysis using the open-access tool *STRING database* (STRING, https://string-db.org, accessed 13 September 2024) [24]. We developed a virtual overview of our research findings, as depicted in Figure 7A. Our analysis revealed direct connections between SDC3, proto-oncogene tyrosine-protein kinase *Src* (*SRC*), fibroblast growth factor 2 (*FGF2*), and matrix metalloproteinase 7 (*MMP7*). Through *SRC* and *FGF2*, SDC3 was shown to interact with vascular endothelial growth factor receptor 1 (*FLT1*), vascular endothelial growth factor receptor 2 (*KDR*), vascular endothelial growth factor B (*VEGFB*), and vascular endothelial growth factor C (*VEGFC*). Indirect connections between SDC3 and cadherin-1 (*CDH1*) through *SRC*, *FGF2*, and *MMP7* were further established. Moreover, multiple interactions between SDC3 and the matrix metalloproteinases-1, -2, -9, and -14 (*MMP1*, *MMP2*, *MMP9*, and *MMP14*), as well as the metalloproteinase inhibitor-2 (*TIMP2*) and -4 (*TIMP4*), were visualized. Figure 7B depicts the gene ontology (GO) of the interconnections between SDC3 and the presented markers in Figure 7A. The *biological processes* category included mechanisms like cell migration, transmembrane receptor protein tyrosine kinase signaling, vascular endothelial growth receptor signaling, chemotaxis, vascular endothelial growth factor signaling, tube morphogenesis, and angiogenesis (Figure 7B, blue). In terms of *molecular function*, SDC3 was linked to *metalloendopeptidase-, vascular endothelial factor receptor-*, *chemoattractant-*, *protein tyrosine kinase-*, *transmembrane receptor tyrosine kinase-*, and *serine-type endopeptidase activity*. Furthermore, SDC3 was associated with the functions of *protein binding*, *growth factor receptor binding*, *catalytic activity acting on a protein*, *and integrin binding* (Figure 7B, green). The *cellular component* analysis demonstrated that SDC3 was related to the *extracellular region*, *-space*, and *-matrix*, as well as to other cellular components, such as *cytoplasmic vesicles*, *sorting endosomes*, *platelets alpha granule lumen*, *endomembrane systems*, and *endosomes* (Figure 7B, orange). *KEGG-pathway* analysis revealed interconnections between SDC3 and the *Rap1-signaling*, *focal-adhesion-signaling*, *Ras-signaling*, *Relaxin-signaling*, *MAPK-signaling*, *EGFR-tyrosine-kinase-inhibitor-resistance*, *PI3K-Akt-signaling*, and *adherens-junctions*-pathways. Also was associated with *pathways-related-to-proteoglycans-in-cancer*, *-to-bladder-cancer*, *-to-melanoma*, *-to-fluid-shear-stress-and-atherosclerosis*, and *to-gastric-cancer signaling* (Figure 7C).

## 4. Discussion

Breast cancer is the most frequently diagnosed malignant tumor in women worldwide [1,2]. Its incidence is rising across all regions globally, despite significant advancements in early detection and treatment development. Enhancing therapeutic strategies and identifying reliable predictive and prognostic markers, therefore, remain key priorities in ongoing research [4].

Syndecans are integral cell surface heparan sulfate proteoglycans that influence multiple signaling pathways, regulating cell proliferation, adhesion, motility, angiogenesis, wound repair, and inflammation [13]. In cancer, syndecans can be aberrantly expressed and are implicated in tumorigenesis and metastasis [15]. Among all members of the syndecan family, SDC3 remains the least studied to date, with limited data currently available on its dysregulation in human malignancies [42].

Our in-silico analysis of the *TNM-plot-*, and *The Human Protein Atlas*-database revealed that SDC3 mRNA expression was significantly upregulated in breast cancer tissue, in comparison to normal breast tissue. In breast cancer metastasis, no significant alterations in SDC3 expression were detected, which might suggest that the molecule plays a distinct role in the early and late stages of breast tumor development. Interestingly, the analysis of *The Human Protein Atlas* demonstrated that SDC3 mRNA and protein are mainly localized in the mitochondria and nucleoplasm of human cells. Since a protein’s function is often closely tied to its precise location within tissues, cells, and subcellular structures, we suggest that the spatial distribution of SDC3 may be of relevance to breast tumor biology [19]. Using the *Kaplan–Meier Plotter* online tool, we explored the association between SDC3 and the relapse-free survival (RFS) of breast cancer patients. High SDC3 expression was significantly linked to improved RFS (*n* = 4929), particularly in the HER2-negative, luminal A, luminal B, and basal subtype, as well as in patients receiving neoadjuvant chemotherapy, suggesting a potential stage-specific role for SDC3 in breast cancer metastasis. Supporting this theory, a study on ovarian cancer found that SDC3 RNA expression was higher in primary tumors compared to metastatic sites, further indicating the potential association of the molecule with early-stage cancer progression [16].

SDC3 expression has previously been observed in various cancer cell lines (i.e., bladder cancer, hepatocellular carcinoma, mammary carcinoma, ovarian cancer, pancreatic cancer, prostate carcinoma, renal cell carcinoma, and glioma cell lines) [43], and in cells of the tumor microenvironment (TME), specifically tumor-associated macrophages and endothelial cells [43]. Interestingly, the molecule has been recently identified as a modulator of macrophage function, aiming at supporting a pro-inflammatory and antitumor phenotype. SDC3 defective macrophages were found to exhibit distinctive gene expression patterns, resulting in impaired tumor cell phagocytosis and increased tumor cell proliferation [44].

However, the distinct SDC3 expression patterns in each cancer type and its correlation with cancer progression largely remain unexplored. Previous studies on SDC3 in breast cancer have yielded mixed results [44,45]. Wu et al. (2013) found in an immunohistochemical and in situ hybridization study on 159 breast cancer patients that GFRα1 (GDNF family receptor alpha-1) and GFRα3 (GDNF family receptor alpha-3) (which, like SDC3, bind Artemin/ARTN) were better predictors of breast cancer progression than SDC3 [45]. In contrast, Jiang et al. (2021) included SDC3 in a seven-gene glycolysis-related signature (PGK1, CACNA1H, IL13RA1, SDC1, AK3, NUP43, SDC3) that predicted poorer prognosis in high-risk breast cancer patients in an analysis of *The Cancer Genome Atlas* mRNA profiling datasets [46]. Notably, Kaplan-Meier analysis in their study showed that higher SDC3 expression alone was associated with a better prognosis, in accordance with our study [46]. Therefore, the difference in methodology (immunohistochemistry vs. transcriptomics) and the size of the sample collectives (being substantially higher in the transcriptomic studies) may have accounted for the discrepancies.

Building upon our in-silico findings, we conducted several in vitro experiments using the human breast cancer cell lines MDA-MB-231 and MCF-7. Gene silencing of SDC3 was achieved by two independent siRNA-, and siPool-knockdown approaches. Subsequently, several functional assays were performed to assess the metabolic activity, cell cycle progression, 3D-spheroid formation, and cell migration in both SDC3-depleted and control MDA-MB-231 and MCF-7 cells. Surprisingly, in contrast to the positive prognostic value of high SDC3 expression, SDC3 depletion in breast cancer cells in vitro resulted in a reduction in pathogenetic phenotypes. This may suggest a differential role for cancer cell-autonomous SDC3 and SDC3 in the tumor microenvironment, as the public gene expression datasets are derived from whole tumor containing tumor cells as well as cells of the tumor microenvironment (cancer-associated fibroblasts, infiltrating immune cells, blood vessel-derived endothelial and smooth muscle cells). Indeed, SDC3 expressed by macrophages infiltrating tumor tissue may contribute to tumor cell phagocytosis and decreased tumor cell proliferation, thus exhibiting an antitumoral effect [44].

Moreover, SDC3 mediates localization of TFPI—a molecule endowed with tumor suppressor activity [40,41]—to the surface of endothelial, smooth muscle, and breast cancer cells [41], which may contribute to an antitumoral effect of SDC3 in the breast cancer microenvironment.

With respect to breast cancer cell-autonomous SDC3, our MTT analysis demonstrated that SDC3-depletion in both MDA-MB-231 and MCF-7 cells resulted in a significant reduction in metabolic activity. This finding is supported by a study on ovarian cancer, which reported that SDC3 knockdown significantly reduced cell viability in SKOV3 ovarian cancer cells [16]. We can only speculate if our MTT assay findings align with the analysis of *The Human Protein Atlas*, possibly suggesting a link between SDC3, cellular metabolism, and its mitochondrial localization.

Our cell cycle analysis revealed that siRNA-mediated knockdown of SDC3 caused G1-phase accumulation and reduced S-phase entry, particularly in MDA-MB-231 cells. SDC3 depletion by siPool-transfection did not yield similar effects, possibly due to differences in knockdown efficiency or compensatory mechanisms. We did not observe any effects of SDC3 depletion on apoptosis in our experimental model (results not shown). SDC3 has been previously identified as a regulator of the cell cycle in cerebellar granule cell precursors (CGCPs), where it was shown to interact with sonic hedgehog (Shh) signaling to modulate cell cycle exit and differentiation [47]. Additionally, loss of SDC3 was found to impair the cell cycle progression of satellite cells (SCs), the adult skeletal muscle progenitors, resulting in reduced progenitor cell expansion, delayed onset of differentiation, increased cell death, and diminished self-renewal capacity [48].

Breast cancer progression, treatment resistance, and relapse are greatly influenced by the activities of cancer stem cells (CSCs) [49]. CSCs possess multiple self-renewal and differentiation abilities that support tumor heterogeneity. Previous studies have shown that heparan sulfate proteoglycans can modulate breast stem cell properties [50]. Therefore, we evaluated the effect of SDC3 depletion on the growth of three-dimensional (3D) spheroids in a hanging drop assay, a method considered suitable for studying stem cell biology in vitro [28,29]. SDC3 knockdown in both MDA-MB-231 and MCF-7 cells resulted in altered spheroid morphology and a reduction in spheroid size, compared to the negative controls. This may suggest a potential role for SDC3 in enforcing CSC attributes. SDC3 has been previously shown to play a role in regulating physiological stem cell function as well as cell adhesion [48,51,52]. The molecule has been implicated in bone-marrow-derived-mesenchymal stem cell (MSC) biology [51], in stem cell-related *notch-signaling* during myogenesis [48], and in the differentiation of neural stem cells (NSCs) [52]. In ovarian cancer, a link between SDC3 and the CSC phenotype has already been established [16]. It was demonstrated that SDC3 depletion in SKOV3 and CAOV3 cells led to significant changes in spheroid morphology, along with altered RNA expression of several stemness-related constituents of the *notch-*, *wnt-*, and *hedgehog pathway*. Similarly, breast cancer initiation and progression were found to be influenced by a dysregulated *notch-*, *hedgehog-*, and *wnt-* signaling [53]. Our qRT-PCR analysis revealed that *Gli2* (associated with *hedgehog*-signaling) in MDA-MB-231 cells and *HES1* (associated with *notch*-signaling) in MCF-7 cells were significantly altered following SDC depletion. These findings may suggest that SDC3 plays a role in regulating *notch*- and *hedgehog*-signaling pathways across different cancer types. As a caveat, the observed SDC3-dependent changes in spheroid size may have been influenced by changes in cell viability, which could have acted as a confounder. Both CSC-related and other factors (such as altered Src -dependent signaling) could have contributed to this phenotype.

Syndecans are important mediators of cancer cell migration, due to their ability to bind different growth factors, chemokines, morphogens, and extracellular matrix components [13,15,54]. In our in vitro migration assay, siRNA-mediated SDC3 depletion led to reduced cell migration in both MDA-MB-231 and MCF-7 cells. This effect was statistically significant in MDA-MB-231 cells, but non-significant in MCF-7 cells. Since MCF-7 cells form tight cell–cell junctions and are more differentiated than MDA-MB-231 cells [26], this finding may be attributable to inherent differences in the characteristics of these cell lines. Notably, reduced motility of SDC3-depleted MDA-MB-231 cells was also observed in a wound closure assay in the presence of mitomycin C, excluding a major confounding effect of cell proliferation in this setting. Our observation of a localization of SDC3 protein to focal adhesions, and the reduction in vinculin staining observed in SDC3-depleted cells may point at a role for this proteoglycan in cell–matrix interactions.

Indeed, SDC3 was reported to play a role in cell motility in both inflammatory and neurobiological contexts [55,56,57]. The molecule was found to inhibit leukocyte migration in vitro and to reduce disease severity in animal models of rheumatoid arthritis [58]. Furthermore, SDC3 deficiency has been shown to impair neural cell migration and to perturb the laminar structure of the cerebral cortex during brain development [55]. Interestingly, it was observed that SDC3 interacts with the glial cell line-derived-neurotropic factor family ligands (GFL) with the involvement of *Src* kinase activation, thereby mediating cell spreading and neurite outgrowth [54].

To further investigate the involvement of SDC3 in cell migration, invasion, and epithelial-to-mesenchymal transition (EMT), we examined its role at the molecular level using qRT-PCR analysis. EMT is defined as a cellular program, in which epithelial cells acquire a mesenchymal phenotype that enables cell migration and invasion of secondary sites [59]. Our analysis revealed that SDC3 depletion in both MDA-MB-231 and MCF-7 cells significantly impacted the expression of EMT-markers. SDC3 knockdown resulted in the downregulation of *Twist* in MDA-MB-231 cells, as well as in increased expression of *CDH1* in MCF7 cells. Furthermore, SDC3 depletion significantly upregulated *MMP1*, and downregulated *MMP9* in MDA-MB-231 cells, suggesting a multifunctional role of the molecule in modulating migration and invasion in breast cancer [60,61]. SDC3 has been previously associated with *MMP*-mediated shedding in neurobiological contexts [62]. Predictive algorithms have suggested roles for *MMP2* and *MMP9* at multiple cleavage sites of SDC3; however, the exact mechanisms and protease in SDC3 shedding have not yet been confirmed experimentally [63,64].

Moreover, we aimed to explore the effect of SDC3 on angiogenesis at the molecular level. Syndecans have previously been shown to mediate VEGF-associated signaling and regulate angiogenesis [31,65]. In our qRT-PCR analysis, we observed that SDC3 knockdown upregulated VEGF-A expression in MCF-7 cells. SDC3 has been reported to suppress endothelial cell migration, indicating a potential antiangiogenic function of the molecule [66]. On the other hand, thrombin-cleaved SDC3 fragments were found to induce endothelial hyperpermeability, having pro-angiogenic effects [67]. It has been revealed that hypoxia activates the hypoxia-inducible factor 1-alpha (HIF1α)/vascular endothelial growth factor A (VEGF-A) axis in breast cancer to promote angiogenesis [35]. Moreover, hypoxia-induced angiogenic HIF1α/VEGF-A signaling was related to SDC3 upregulation in hypoxic tumors, which further implicates SDC3 in breast tumor growth and vascular remodeling [43].

To evaluate the underlying mechanisms by which SDC3 influences cell viability, cell-cycle regulation, stemness, and migration in breast cancer, we investigated the effects of SDC3 depletion on the activation of proto-oncogene tyrosine-protein kinase *Src* (*pSRC*, *SRC*). *Src* has been previously linked to SDC3 function in a neurobiological context [36]. Our results showed that *Src* activation was inhibited in MCF-7 and MDA-MB-231 cells when it was combined with TFPI treatment. Indeed, TF/FVIIa has been shown to activate c-Src in endothelial cells [37], which is consistent with the inhibitory effect of TFPI in our system. Moreover, the previous finding that SDC3 mediates cell surface localization of TFPI in breast cancer and vascular cells [41] may have contributed to this impact on *Src* signaling. *Src* has been shown to be involved in multiple signaling pathways, such as *PI3K/AKT* and *HIF1α*, and to impact tumor angiogenesis, metabolism, and metastasis [38,39,68,69]. Of note, *Src* has also been associated with activating signal transduction pathways leading to the expression of *HIF* and its downstream genes, providing a molecular mechanism for tumor cells to adapt to a hypoxic environment [39]. *HIF* regulates several pro-tumorigenic genes, including those involved in angiogenesis (i.e., *VEGFA*, *IL-8*), glycolytic cellular metabolism (i.e., *LDHA*), extracellular matrix remodeling (i.e., *MMPs*), epithelial-to-mesenchymal transition (i.e., *CDH1*), metastasis (i.e., *TWIST1*), and cell motility and adhesion (i.e., *integrins*) [70].

To summarize our key research findings, we developed a virtual overview of the signaling pathways affected by SDC3 using the *STRING* database. Our analysis revealed direct connections between SDC3, proto-oncogene tyrosine-protein kinase *Src* (*SRC*), fibroblast growth factor 2 (*FGF2*), and matrix metalloproteinase 7 (*MMP7*). Further indirect associations were established between SDC3 and vascular endothelial growth factor receptor 1 (*FLT1*), vascular endothelial growth factor receptor 2 (*KDR*), vascular endothelial growth factor B (*VEGFB*), vascular endothelial growth factor C (*VEGFC*), cadherin-1 (*CDH1*), as well as matrix metalloproteinases-1, -2, -9, and -14 (*MMP1*, *MMP2*, *MMP9*, and *MMP14*).

## 5. Conclusions

Our findings suggest that SDC3 may influence critical cancer hallmarks associated with breast tumorigenesis and cancer progression. We propose that SDC3 may regulate the activity of the proto-oncogene tyrosine-protein kinase Src (*SRC*) in the context of the TF pathway, as well as other genes involved in crucial breast cancer signaling pathways, thereby affecting human breast cancer cell behavior. This molecular analysis may provide a mechanistic explanation for the functional changes observed upon SDC3 depletion. Future research should further elucidate SDC3-related signal transduction pathways in the context of breast cancer and a possible antitumoral effect of SDC3 of the tumor microenvironment, which may contribute to its positive predictive value in transcriptome-based survival analysis. Based on our findings and the existing literature, an association between the molecule and the previously described pathways appears likely. Further investigation, using advanced preclinical models, could provide valuable insights into its potential as a therapeutic target or biomarker in breast cancer.

## Figures and Tables

**Table 1:** SDC3 protein expression in breast tumor samples 1–8.

Sample	Patient ID	Staining	Intensity	Quantity	Location
1	#2898	medium	moderate	>75%	cytoplasmic, membranous
2	#3257	medium	moderate		
3	#2392	medium	moderate		
4	#1939	medium	moderate		
5	#2805	low	weak		
6	#1874	low	weak		
7	#2428	low	weak		
8	#2174	low	weak		

**Table 2:** Correlation between SDC3 RNA expression and RFS of breast cancer patients.

Classification	Status	Cases	Hazard Ratio	*p* Value
All breast cancer patients		4929	0.75 (0.68–0.83)	**Log-rank *p* = 1.7** ** × ** **10^−8^**
Estrogen receptor (ER)	Positive	2561	0.87 (0.75–1.02)	Log-rank *p* = 0.079
	Negative	796	0.81 (0.64–1.03)	Log-rank *p* =0.086
Progesterone receptor (PR)	Positive	926	0.95 (0.71–1.26)	Log-rank *p* = 0.72
	Negative	925	0.91 (0.72–1.14)	Log-rank *p* = 0.41
Her2	Positive	882	0.85 (0.68–1.06)	Log-rank *p* = 0.15
	Negative	4047	0.73 (0.65–0.81)	**Log-rank *p* = 3.6** ** × ** **10^−8^**
ER, PR, Her2	Negative	392	0.9 (0.63–1.29)	Log-rank *p* = 0.56
St. Gallen subtype	Luminal A	2277	0.77 (0.65–0.91)	**Log-rank *p* = 0.0016**
	Luminal B	1419	0.71 (0.59–0.85)	**Log-rank *p* = 0.00015**
	Her2 positive	315	0.9 (0.64–1.29)	Log-rank *p* = 0.6
	Basal	846	0.72 (0.58–0.9)	**Log-rank *p* = 0.004**
PAM50 subtype	Luminal A	1809	0.88 (0.72–1.08)	Log-rank *p* = 0.23
	Luminal B	1353	0.83 (0.7–0.99)	**Log-rank *p* = 0.038**
	Her2 positive	695	0.86 (0.68–1.1)	Log-rank *p* = 0.23
	Basal	953	0.69 (0.56–0.86)	**Log-rank *p* = 7** ** × ** **10^−4^**
Lymph node	Positive	1656	0.97 (0.82–1.15)	Log-rank *p* = 0.75
	Negative	2368	0.74 (0.63–0.87)	**Log-rank *p* = 0.00027**
Grade	1	397	1.12 (0.73–2)	Log-rank *p* = 0.47
	2	1177	0.74 (0.59–0.92)	**Log-rank *p* = 0.0057**
	3	1300	0.88 (0.73–1.06)	Log-rank *p* = 0.16
Treatment with chemotherapy	Neoadjuvant	402	0.6 (0.41–0.88)	**Log-rank *p* = 0.009**
	Adjuvant	458	0.93 (0.67–1.3)	Log-rank *p* = 0.68
Treatment with endocrine therapy		867	0.86 (0.66–1.12)	Log-rank *p* = 0.25
Treatment with chemotherapy and endocrine therapy		510	1.22 (0.81–1.83)	Log-rank *p* = 0.34

Bold values indicate statistically significant differences.

## Data Availability

The data are contained within the article.

## Supplementary Materials

The following supporting information can be downloaded at: https://www.mdpi.com/article/10.3390/cells14201612/s1, Table S1. SYBR Green PCR primer sequences used in this study. Supplementary Figure S1 SDC3 depletion affects the RNA expression of several target genes associated with relevant breast cancer signaling pathways. The gene expression of syndecan-1 (Sdc1) and syndecan-4 (Sdc4) in SDC3 depleted cells was evaluated to determine potential compensatory expression changes. The expression of target genes associated with notch-signaling (Notch1, HES1), wnt-signaling (WNT5A, TCF7L1), hedgehog-signaling (Gli1, Gli2, Gli3), epithelial-to-mesenchymal-transition signaling (Twist, Snail1, Snail2, CDH1, Vimentin), matrix–metalloproteinase signaling (MMP1, MMP2, MMP9), vascular–endothelial growth factor signaling (VEGF-A), interleukin-signaling (IL-8), collagen-signaling (COL4A2), and CD44-signaling (CD44) was further investigated. *p* ≤ 0.05 was considered statistically significant (*), while *p* ≤ 0.01 was described as very significant (**), and *p* ≤ 0.001 as highly significant (***). The graphs depict mean values, with error bars indicating the standard error of the mean (SEM). Three independent experimental replicates were included (*n* = 3).
