# Supplementary material for: The Role of the Cell Surface Heparan Sulfate Proteoglycan Syndecan-3 in Breast Cancer Pathophysiology
Source: Cells. 2025 Oct 17;14(20):1612. doi: 10.3390/cells14201612 (PMC12562340; doi:10.3390/cells14201612)
Supplement: Supplementary file 1 [file cells-14-01612-s001.zip › cells-3729915-supplementary.pdf]

Article: The Role of the Cell Surface Heparan Sulfate Proteoglycan Syndecan-3 in Breast Cancer Pathophysiology.

Supplementary Materials

Figure SI: SDC3 depletion affects the RNA expression of several target genes associated with relevant breast cancer signaling pathways.

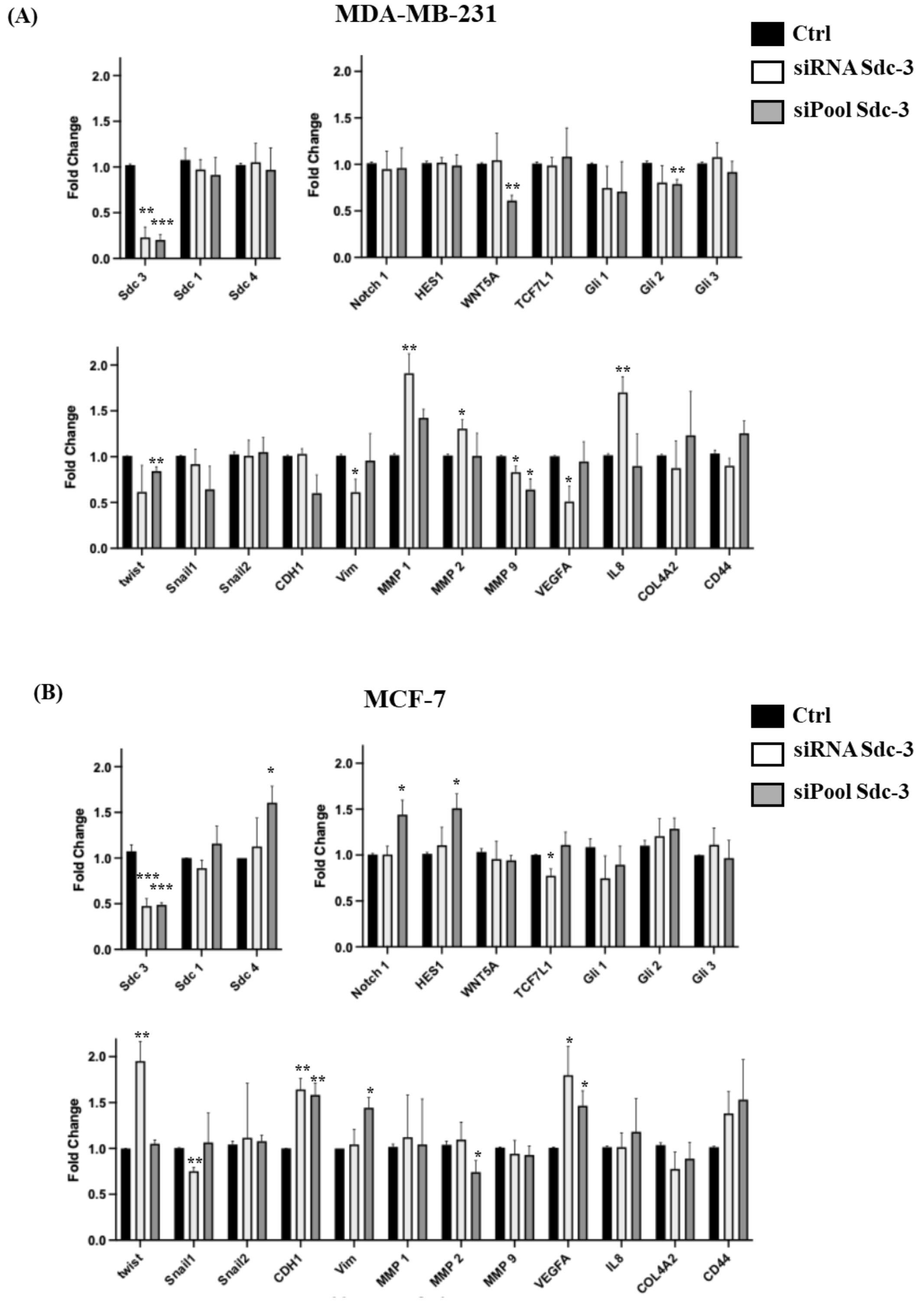

Table SI: SYBR Green PCR primer sequences used in this study.

Gene	Primer sequence (5´-3´) Forward	Primer sequence (5´-3´) Reverse
**Actin**	CAA AGA CCT GTA CGC CAA CAC	CAT ACT CCT GCT TGC TGA TCC
**Sdc1**	AGG ACG AAG GCA GCT ACT CCT	TTT GGT GGG CTT CTG GTA GG
**Sdc3**	CTC CTG GAC AAT GCC ATC GACT	TGA GCA GTG TGA CCA AGA AGGC
**Sdc4**	TGT CCA ACA AGG TGT CAA TG	GTT TCT TGC CCA GGT CAT AG
**CDH1**	CAA AGC CCA GAA TCC CCA AG	CAC ACC TGG AAT TGG GCA AA
**CD44**	GCC CAT TGT TCA TTC TTG TGC T	AAA CCA GAG GAA GGG TGTGCT C
**COL4A2**	CAG AAG TGG AGA CCT TTC TAG ACA TCA	GGG TTA AAT CTC AGG GAC AAC GTG
**Gli1**	TTC CTA CCA GAG TCC CAA GT	CCC TAT GTG AAG CCC TAT TT
**Gli2**	GTC AGA GCC ATC AAG ACC GAGA	GCA TCT CCA CGC CAC TGT CATT
**Gli3**	TCA GCA AGT GGC TCC TAT GGT C	GCT CTG TTG TCG GCT TAG GAT C
**HES1**	GTG AAG CAC CTC CGG AAC	CGT TCA TGC ACT CGC TGA
**IL8**	GAG AGT GAT TGA GAG TGG ACC AC	CAC AAC CCT CTG CAC CCA GTTT
**MMP1**	CGA CTC TAG AAA CAC AAG AGC AAGA	AAG GTT AGC TTA CTG TCA CAC GCTT
**MMP2**	AGC GAG TGG ATG CCG CCT TTAA	CAT TCC AGG CAT CTG CGA TGAG
**MMP9**	ATT TCT GCC AGG ACC GCT TC	CTC AGG CAC TGC AGG ATGT
**Notch1**	GGT GAG ACC TGC CTG AATG	GTT GGG GTC CTG GCA TC
**Snail1**	CGA GCC CAG GCA GCT ATT TC	CCC GAC AAG TGA CAG CCA TT
**Snail2**	ATC TGC CAG ACG CGA ACT CA	GGC AAC CAG ACA ACC GAC AT
**TCF7L1**	AAG GTG CCT GCC ACT TCC TC	CCT GCC ACT CTG GGA TTG TG
**Twist**	GCG GCC AGG TAC ATC GAC TT	TGC AGC TTG CCA TCT TGG AG
**VEGFA**	TTG CCT TGC TGC TCT ACC TCCA	GAT GGC AGT AGCT GCG CTG ATA
**Vim**	TCA GCA TCA CGA TGA CCT TGAA	CTG CAG AAA GGC ACT TGA AAGC
**WNT5A**	TCG TTA GCA GCA TCA GTC CACA	GAC CTG TGC CTT CGT GCC TA
